# Use of fulvic acid-like compounds from pulp-derived black liquor for enhancing the selenium content of peanut buds

**DOI:** 10.1186/s12870-022-03903-3

**Published:** 2022-11-28

**Authors:** Feng Ding, Xiaofeng Wei, Yuanren Dao, Fei Zhao, Ruiming Wang, Piwu Li

**Affiliations:** State Key Laboratory of Biobased Material and Green Papermaking (LBMP), College of Bioengineering, Qilu University of Technology (Shandong Academy of Sciences), Jinan, 250353 People’s Republic of China

**Keywords:** Fulvic acid-like substances, Peanut sprout, Selenite tolerance and selenium accumulation, Edible quality and food safety

## Abstract

**Background:**

Cleaner production involving the extraction of useful material from the black liquor by-product of straw pulp would be environmentally beneficial and would permit increased wastewater usage.

**Results:**

The fulvic-acid-like components of pulp black liquor (PFA) with molecular weights below 10 kDa were isolated. The chemical and physiological characteristics of PFAs were investigated. Selenite can enhance the selenium nutrition level of crops, but excessive selenite may be toxic to plant growth. In order to explore how to increase selenite tolerance and selenium accumulation in peanut, the effects of PFA on selenium-associated properties in peanut seedlings were examined by growing seedlings with sodium selenite (0, 5, 15, and 25 mg·L^− 1^ Na_2_SeO_3_, 15 mg·L^− 1^ Na_2_SeO_3_ solution containing 60 mg-C/L PFA, and 25 mg·L^− 1^ Na_2_SeO_3_ containing 60 mg-C/L PFA).

**Conclusion:**

The results showed that with 15 mg·L^− 1^ Na_2_SeO_3_, PFA significantly increased both the total and hypocotyl fresh weight of the seedlings but reduced the fresh weight of the root. PFA also effectively promoted the conversion of Se from inorganic to organic compounds in the root and hypocotyl, increased the soluble total sugar and soluble protein contents of the hypocotyl, and thus improved the edible quality and food safety of the selenium-enriched peanut buds. The results suggest that PFA can be used as an innovative bio-based substance for selenium-enriched sprout vegetable production.

## Introduction

Black liquor is a by-product in the paper industry and its treatment is essential for cleaner production. Cost-effective reclamation of the organic constituents in black liquor reduces potentially deleterious environmental effects of the waste material. Yao investigated the fulvic-acid-like components of black liquor (PFA), examining their chemical structures and effects on physiology and growth. These authors observed that salt-stressed rice seedlings grew well in the presence of low molecular weight (below 5 kDa) PFAs but less so in the presence of PFAs with higher molecular weights (above 10 kDa) [1]. PFAs from pulp black liquor were also reported to have the same growth-promoting activities in rice seed germination as compounds from leonardite [2].

Selenium (Se) is an essential mineral, playing vital roles in various metabolic processes, and Se deficiency can cause multiple health issues [3]. It has been found that organic compounds are a better source of Se than their inorganic counterparts due to their reduced toxicity [4, 5]. As plants convert Se from inorganic to organic forms, they are useful for the provision of organic Se compounds. Se levels in crop plants can be increased in several ways. The principal method is the use of Se-containing fertilizer [6]. However, not all plants are able to accumulate Se effectively; while Se-accumulating plants may be highly efficient in this respect [7], the majority are non-accumulators and may even be sensitive to high Se concentrations, resulting in damage such as poor growth and withered leaves [8]. In plants, Se toxicity is thought to be the consequence of the over-production of nonspecific selenoproteins, although recent evidence has suggested that Se-induced oxidative stress may also be responsible [9].

Peanuts (*Arachis hypogaea* L.) are grown throughout the world and are not Se hyperaccumulators [10]. Application of selenite to the soil has been shown to elevate the organic Se content of the peanut kernel, providing a useful source of organic Se for human nutrition [11]. Peanut sprout is a kind of sprout vegetable whose seeds germinate and grow into edible in a short time. However, the selenium content of peanut sprouts is lower than that of other selenium rich sprouts such as soybean sprouts and mung bean sprouts, and the proportion of organic selenium needs to be further improved to enhance its edible safety [12, 13]. If the selenium concentration in the culture medium is further increased, it will lead to excessive production of non-specific selenoproteins in plant tissues and peroxidation stress, resulting in toxicity to plants. Therefore, it is necessary to explore new methods of Se enrichment.

Fulvic acid (FA) is known to enhance plant growth as it has a low molecular weight [14], is rich in functional groups, and is able to pass through cell membranes with ease [15]. FA can induce changes in the primary and secondary metabolic pathways of plants associated with abiotic stress tolerance. PFA was similar to the FA extracted from leonardite (non-renewable resource). They contained similar elemental compositions and functional groups [2]. However, the compounds in paper mill effluents tend to be larger and thus lack the physiological activities necessary for agricultural utilization [16–18]. Here, we extracted PFAs from the spent liquor of ammonium sulfite pulping. After fractionation by ultrafiltration, PFAs of lower molecular weight (below 10 kDa) were used to investigate their use in the production of Se-enriched peanut sprouts under selenite stress.

## Results and discussion

### Elemental analyses

The elemental contents and atomic ratios of the pulp black liquor (PBL) and PFA are shown in Table 1. The PFA contained less C, N, H, S, and more O than PBL. The H/C ratio was inversely proportional to the aromaticity of a compound and the O/C ratio is directly proportional to the numbers of oxygen-containing functional groups such as carboxyl groups. Fulvic acid-like compounds have higher H/C and O/C ratios than other compounds in the PBL and thus are less aromatic and contain more oxygen-containing functional groups.

**Table 1:** Elemental composition and atomic ratio of PBL and PFA

Sample	Elemental composition					Atomic ratio	
	N (%)	C (%)	H (%)	S (%)	O (%)	H/C	O/C
PBL	8.74	31.66	3.94	12.70	32.81	1.49	0.78
PFA	0.24	14.51	1.23	0.17	44.32	1.02	2.29

### FT-IR spectroscopy

**Fig. 1:**
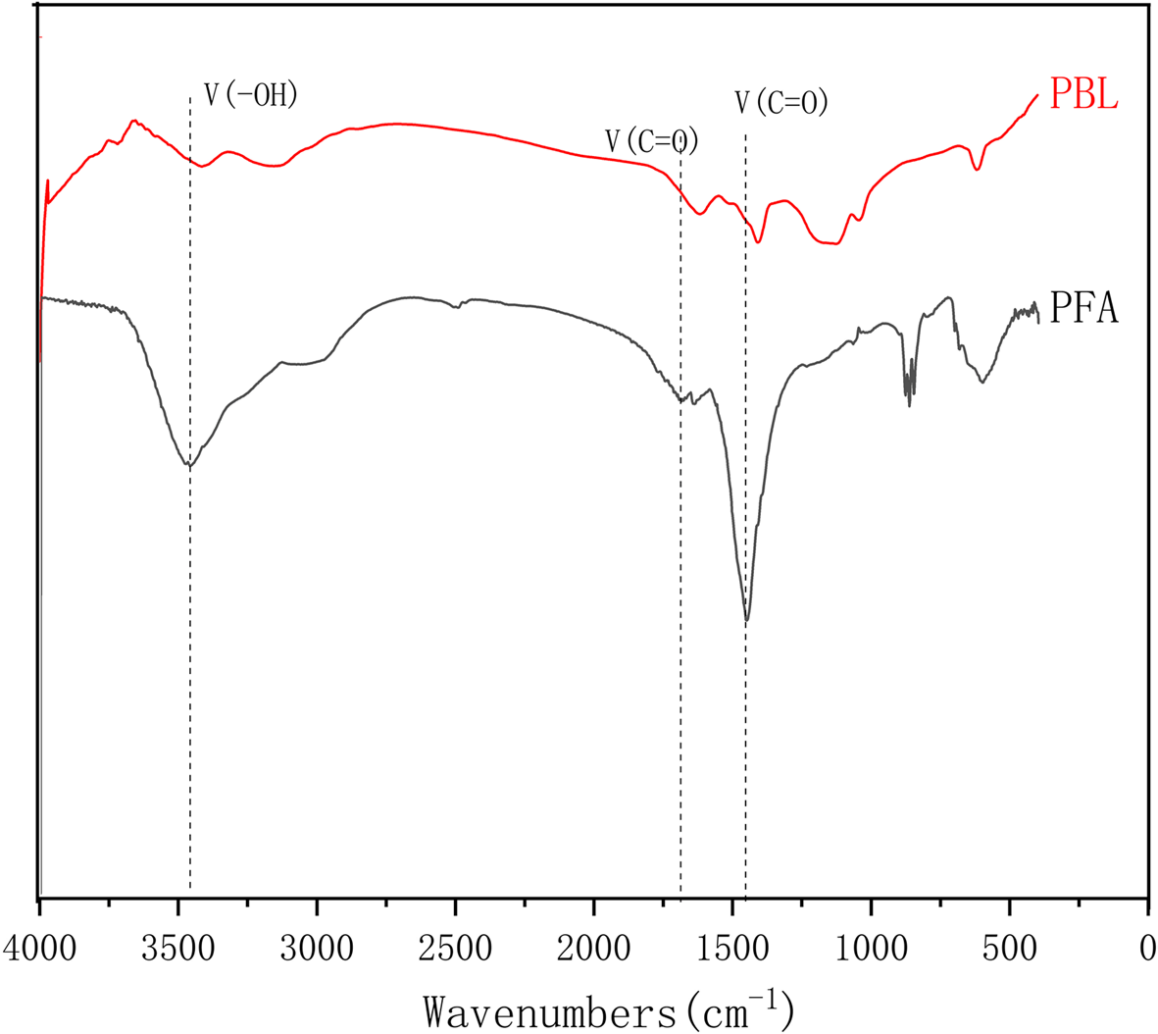
Infrared spectra of PBL and PFA. PBL, Pulp black liquor; PFA, fulvic acid-like in paper mill effluents

The infrared spectra of the PBL and PFA are illustrated in Fig. 1. Overall, a broad band in the region of 3400 cm^− 1^ resulted from both O-H stretching in hydroxyl groups derived from phenol or carboxyl groups. Bands in the region of 2900 cm^− 1^ were the consequence of both symmetric and asymmetric stretching vibrations in aliphatic C-H bonds in ethyl and methyl groups. The band seen at 1710 cm^− 1^ was the result of C=O stretching vibrations in carboxyl groups or derived from additional carbonyl components [19]. A substantial band at 1630 cm^− 1^ was caused by C=C vibrations in aromatic structures conjugated with C=O. PFA samples showed a band at 1465 cm^− 1^, resulting from C-H stretching in aliphatic ethyl and methyl moieties. Thus, more hydroxyl, carboxyl, and small active groups were present in PFA compared with PBL.

### PFA enhanced seedling growth under selenite stress

**Fig. 2:**
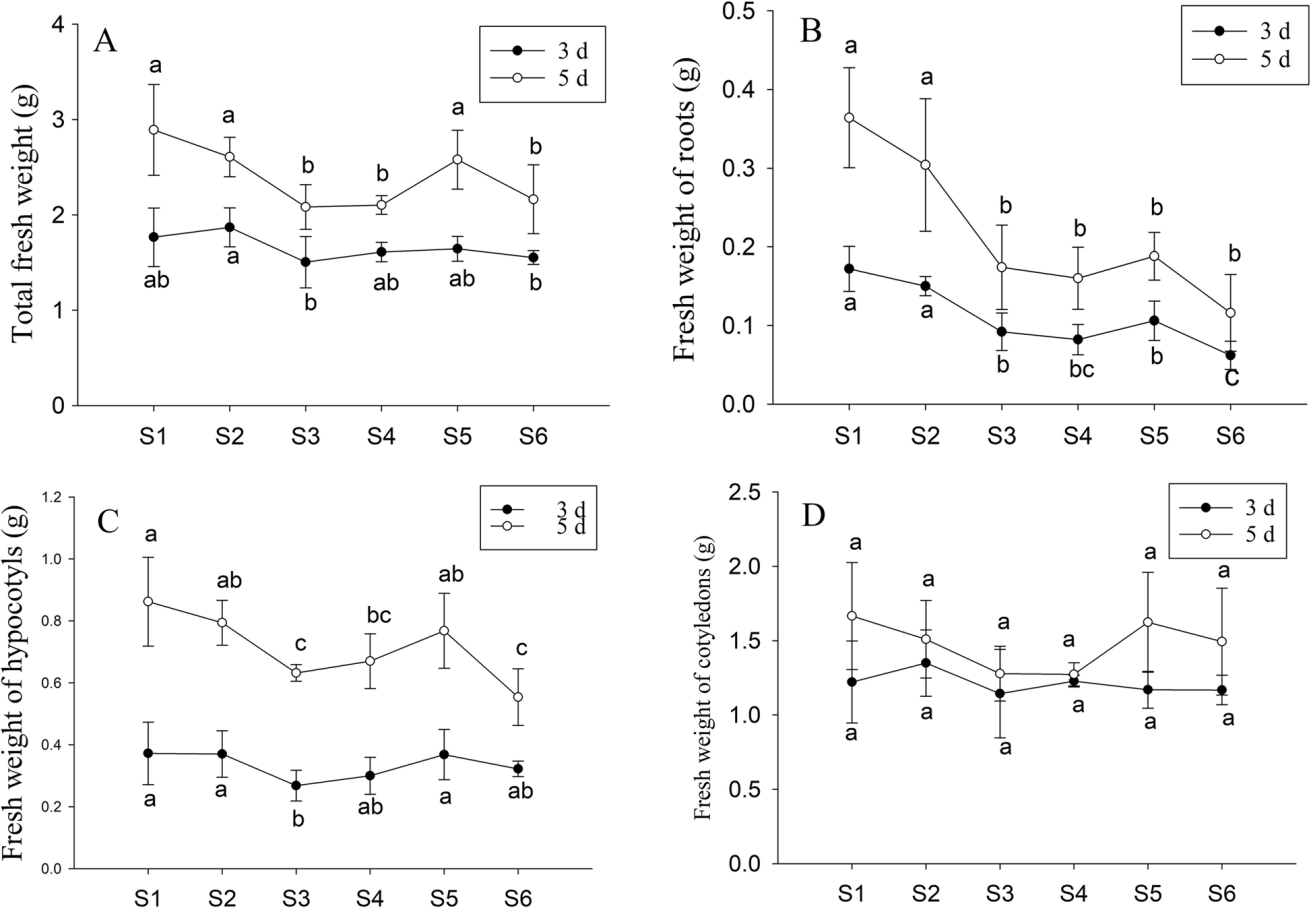
Effects of PFA on the fresh weight of peanut (*Arachis hypogaea* L.) seedlings treated with different selenite concentrations on the 3rd day and 5th day after germination (S1-S6: 0, 5, 15, and 25 mg·L^− 1^ Na_2_SeO_3_, 15 mg·L^− 1^ Na_2_SeO_3_ solution containing 60 mg-C/L PFA, and 25 mg·L^− 1^ Na_2_SeO_3_ containing 60 mg-C/L PFA. The same as below). Letters above the bars indicate significant differences (*P* < 0.05). Vertical bars represent ± SD (*n* = 5). **A** Total fresh weight, **B** Fresh weight of roots, **C** Fresh weight of hypocotyls, **D** Fresh weight of cotyledons

There was a gradual decrease in the total fresh weight, root fresh weight, and hypocotyl fresh weight of the peanut seedlings in response to increasing Na_2_SeO_3_ concentrations, while the cotyledon fresh weight did not change significantly; The total fresh weight of peanut seedlings treated with S3 and S4 decreased by 27.9 and 27.2%, respectively, compared with S1. In seedlings treated with 15 mg·L^− 1^ Na_2_SeO_3_, the addition of PFA (S5) significantly increased both the total and hypocotyl fresh weights by 23.8 and 21.5%, respectively, compared with S3. The addition of PFA did not affect the fresh weights of the roots and cotyledons. Treatment with 15 mg·L^− 1^ Na_2_SeO_3_ did not significantly alter either the total or hypocotyl fresh weights after the addition of PFA (S5) compared with the control (S1), although the root fresh weight decreased by 56%. When PFA (S6) was added to seedlings treated with 25 mg·L^− 1^ Na_2_SeO_3_, the total and hypocotyl fresh weights did not change in comparison with S4 but were significantly reduced compared with the control (S1). These results indicate that in 15 mg · L^− 1^ Na_2_SeO_3_ treatment, the addition of PFA can significantly improve the total fresh weight of peanut seedlings, which has no significant difference with the control (Fig. 2).

**Fig. 3:**
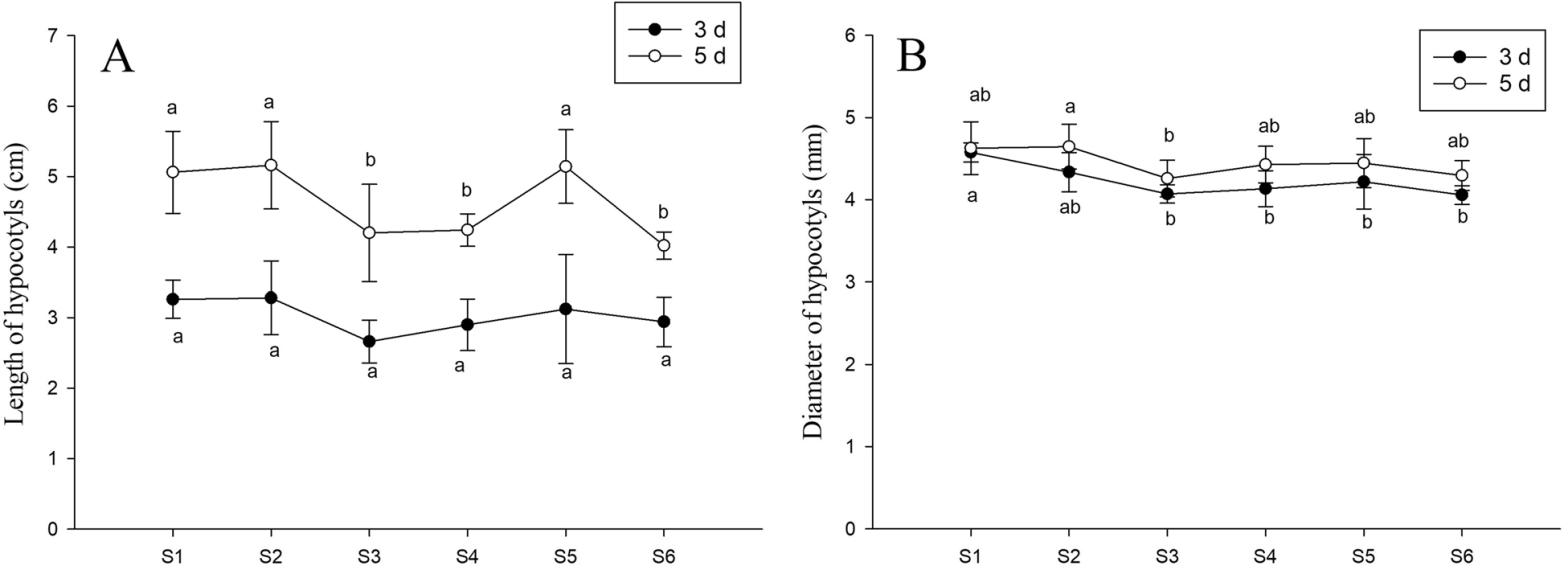
Effects of PFA on the morphology of peanut (*Arachis hypogaea* L.) seedlings treated with different selenite concentrations. Letters over the bars indicate significant differences (*P* < 0.05). Vertical bars represent ± SD (*n* = 5). **A** Length of hypocotyls, **B** Diameter of hypocotyls

The diameters of the peanut hypocotyl did not change significantly with the different treatments; however, the hypocotyl lengths did decrease significantly with increasing concentrations of Na_2_SeO_3_. The hypocotyl lengths in peanuts treated with S3 and S4 decreased by 9.9 and 12.3%, respectively, compared with S1. When PFA was included with 15 mg·L^− 1^ Na_2_SeO_3_ (S5), the hypocotyl length increased by 12.7% compared with S3 while with the Na_2_SeO_3_, with no differences seen between the Na_2_SeO_3_ treatment and control. With 25 mg·L^− 1^ Na_2_SeO_3_ treatment, no significant differences were observed in the hypocotyl length of peanuts treated with PFA (S6) and without PFA (S4), which were significantly reduced in comparison with the control (S1). This indicates that the inclusion of PFA with 15 mg·L^− 1^ Na_2_SeO_3_ significantly increased the total fresh weight of peanut seedlings, which may be due to the increased length of the peanut hypocotyl (Fig. 3).

High Se concentrations have been found not only to reduce the lengths of the hypocotyl and root but also to adversely affect lateral roots in a dose-dependent manner [20]. We found that PFA mitigated the growth-inhibiting effects of Se and that the increase in the total fresh weight of the seedling was mainly due to increases in the weight of the hypocotyl. The addition of PFA, however, did not affect the fresh weight of roots. Moderate applications of humus improve both plant growth and metabolism, leading to increased root lengths and densities, while high concentrations of humic components reduce root growth [1, 21]. Increases in the weight of the hypocotyl and decreases in the weight of the root imply increased weight of the edible part of the peanut bud, promoting the nutritional quality of the peanut bud.

### PFA enhanced the conversion of inorganic to organic se in peanut seedling

**Fig. 4:**
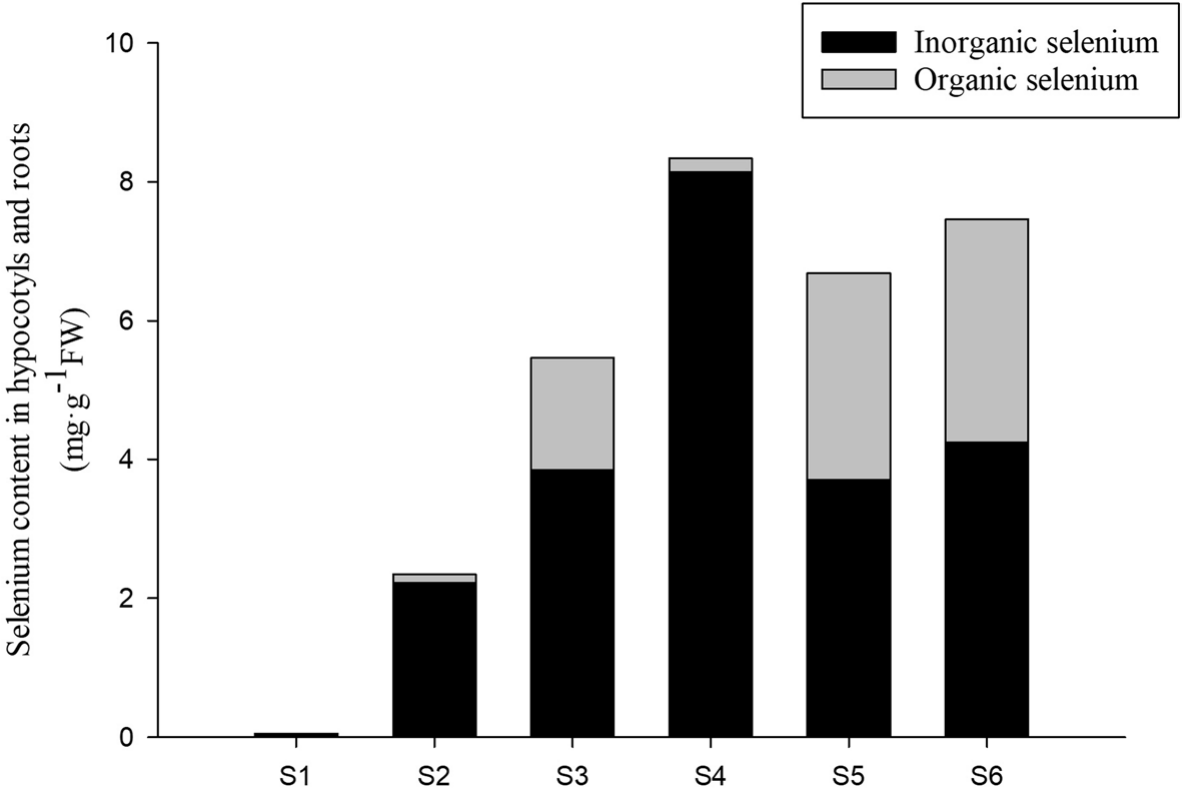
Selenium contents in roots and hypocotyls of the peanut (*Arachis hypogaea* L.) seedling treated with different selenite concentrations on the 6th day after germination

The total Se content of the peanut bud hypocotyl and root increased with increasing Na_2_SeO_3_ concentration, seen as a gradual increase in the content of inorganic Se while the organic Se content increased to a maximum with treatment with 15 mg·L^− 1^ Na_2_SeO_3_ and then decreased. PFA in conjunction with 15 mg·L^− 1^ Na_2_SeO_3_ treatment (S5) resulted in an increase of 22.3% of the total Se content of the hypocotyl in comparison with treatment without PFA (S3); this included an increase of 84.8% in organic Se concentration while the inorganic Se content did not change significantly. Interestingly, under the treatment of 25 mg·L^− 1^ Na_2_SeO_3_, while the total Se content in the hypocotyl did not change significantly after the addition of PFA, the organic Se content increased 15.1-fold together with a decrease of 47.8% in the content of inorganic Se (Fig. 4). These results show that the addition of PFA promoted the conversion of inorganic to organic Se in roots and hypocotyls. As organic Se compounds have lower toxicity than their inorganic counterparts, this implies elevated nutritional safety of the Se-enriched peanut sprouts.

Liu and Wang found that humic acid (HA) can promote selenite absorption by wheat under hydroponic conditions [22]. Fulvic acid (FA) has a simple structure and, when combined with Se, can be mineralized to available Se compounds that are easily absorbed and metabolized by plants, such as Se (IV), Se (VI), and low-molecular-weight organic Se compounds [23, 24]. HA is a large aromatic compound with a stable macromolecular structure that is not easily degraded and is thus not readily available for absorption by plants. Organic Se can be divided into potentially bioavailable Se (hydrophilic organic acid-bound Se and fulvic acid-bound Se) and non-bioavailable Se (HA-bound Se) [25–27]. In the present study, compared with PBL, PFA was less aromatic and contained greater numbers of oxygen-containing functional groups (Table 1), which would be conducive to combining with Se to form bioavailable Se compounds.

The absorption of Se by plants depends on sulfur transporters, an energy-intensive process that operates against an electrochemical potential gradient found that the characteristics of Se-sulfur interactions in tobacco differed with different concentrations of Se and sulfur [28, 29]. Under low-sulfur conditions, Se accumulated in response to increasing sulfur concentrations, with Se and sulfur functioning synergistically. Under high-sulfur conditions, however, Se accumulation decreased in correspondence with increased sulfur concentration, with the two elements behaving antagonistically. Here, it was observed that under 15 mg·L^− 1^ Na_2_SeO_3_ treatment, when PFA was added (S5) the hypocotyl contents of both total and organic Se were significantly raised, possibly linked to the presence of low molecular weight (below 10 kDa) PFA and sulfur in the culture medium. The content of protein-bound Se in the peanut is reported to be high, accounting for 69.29% of the total Se content [30]. The observed increase in organic Se content after the addition of PFA may be related to elevated cysteine and methionine concentrations, which help to synthesize seleno-amino acids.

### PFA activated antioxidant enzymes in peanut seedlings treated with moderate selenite concentrations

**Fig. 5:**
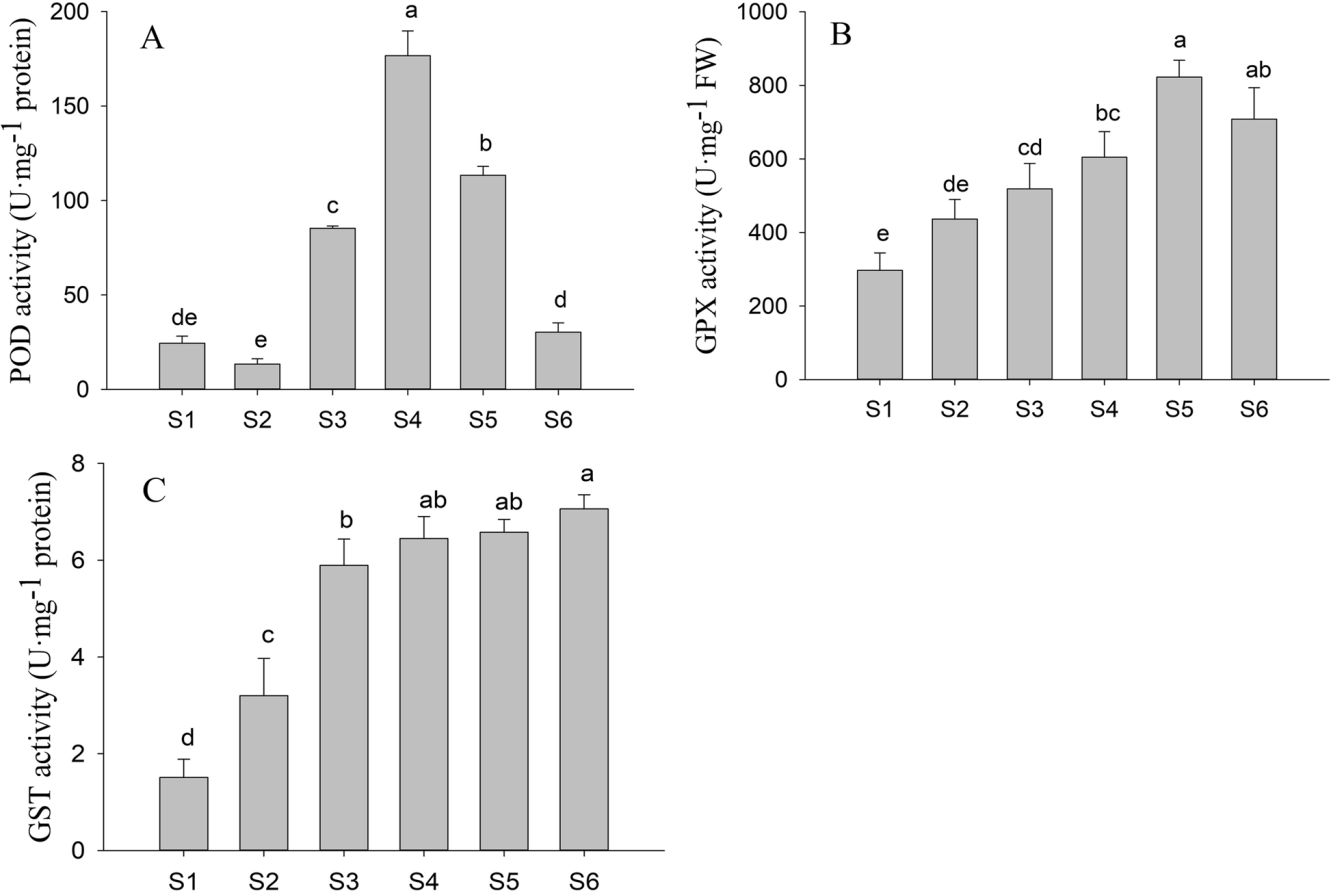
Effects of PFA on the activities of antioxidant enzymes in hypocotyls of peanut (*Arachis hypogaea* L.) seedlings treated with different selenite concentrations on the 6th day after germination. **A** Peroxidase activity, **B** GSH-Px activity, **C** Glutathione S-transferase activity. Letters over the bars indicate significant differences (*P *< 0.05) Vertical bars represent ± SD (*n* = 5)

Increasing Na_2_SeO_3_ concentrations resulted in increased activity of the antioxidant enzymes POD, GPX, and GST in the hypocotyl of the peanut bud (S1-S4). Interestingly, the addition of PFA to seedlings treated with 15 mg·L^− 1^ Na_2_SeO_3_ (S5) resulted in increases in POD and GPX activities of 33.0 and 58.6%, respectively, with no significant effect on GST activity. Under 25 mg·L^− 1^ Na_2_SeO_3_ treatment, the addition of PFA reduced POD activity by 82.8% with no observed change in the activity of GST (Fig. 5). Yao found that FA and FA-like substances at appropriate concentrations enhanced rice resistance to salt stress by upregulating the levels of antioxidant enzymes and thus lowering MDA accumulation [1]. The present study shows that the addition of PFA in appropriate concentrations significantly elevated both POD and GPX activity, thus improving the plant’s ability to scavenge free radicals. Xu found that FA influenced Se incorporation into POD isozymes. At low Se concentrations, FA prevented Se incorporation into POD, while at high Se concentrations, Se incorporation was promoted. This may be the reason why the addition of PFA under moderate Se concentrations enhanced POD activity, with the opposite effect seen under high-Se conditions [31].

### Effects of PFA on the activities of enzymes in the ascorbate−glutathione cycle

**Fig. 6:**
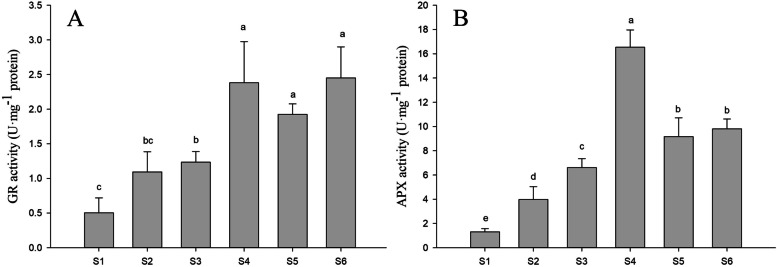
Effects of PFA on activities of enzymes of the ascorbate−glutathione cycle in hypocotyls of peanut (*Arachis hypogaea* L.) seedlings treated with different selenite concentrations on the 6th day after germination. **A** Glutathione reductase activity, **B** Ascorbate peroxidase activity. Letters over the bars indicate significant differences (*P* < 0.05). Vertical bars represent ± SD (*n* = 5)

Increased Na_2_SeO_3_ concentrations resulted in increasing GR and APX activities in the peanut germ hypocotyl (S1-S4). Addition of PFA to seedlings treated with 15 mg·L^− 1^ Na_2_SeO_3_ led to significant increases of 55.9 and 38.7% in GR and APX activity, respectively, in comparison to seedlings not treated with PFA. With 25 mg·L^− 1^ Na_2_SeO_3_, APX activity decreased by 40.7% after PFA addition, while no effect was seen on GR activity (Fig. 6). The ascorbate-glutathione cycle is responsible for the maintenance of cell homeostasis through the scavenging of free radicals [32]. APX reduces H_2_O_2_ to water through the action of ascorbic acid, leading to the formation of an ascorbyl radical and oxidized ascorbate [33] followed by the regeneration of ascorbic acid and the production of GSSG [32, 34]. GSSG is then reduced by GR to maintain normal GSH levels [35]. GSH is responsible for the regeneration of ascorbic acid and GR maintains the ascorbic acid level through the ascorbate−glutathione cycle [36]. Both GSH and ascorbic acid are powerful antioxidants as function as electron donors to numerous cellular processes reactions [37]. Here, we observed that the activities of both APX and GR were significantly raised by PFA treatment in conjunction with 15 mg·L^− 1^ Na_2_SeO_3_. This is important because APX and GR play extremely important roles in the ascorbate−glutathione cycle.

### Effects of PFA on the MDA content under selenite stress

**Fig. 7:**
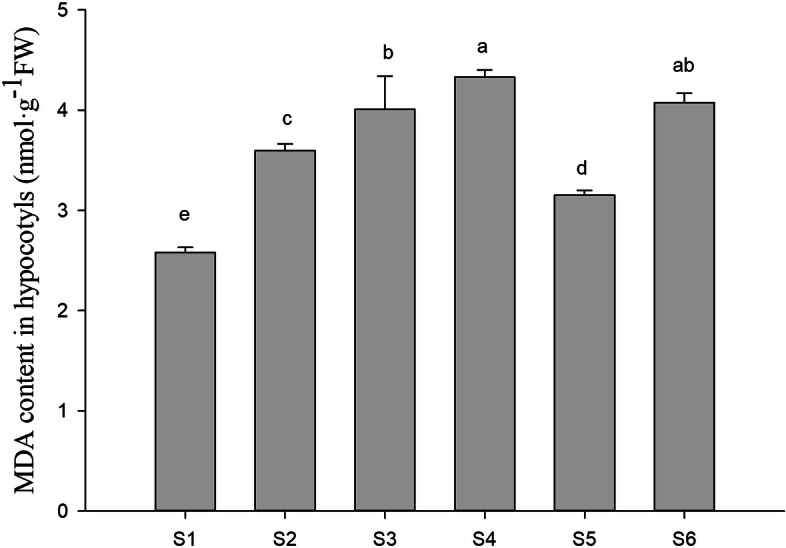
Effects of PFA on the MDA contents in hypocotyls of peanut (*Arachis hypogaea* L.) seedlings treated with different selenite concentrations on the 6th day after germination. Letters over the bars indicate significant differences (*P* < 0.05). Vertical bars represent ± SD (*n* = 5)

The MDA levels in the peanut bud hypocotyls increased gradually with increasing Na_2_SeO_3_ concentration (S1-S4). When PFA was added to seedlings treated with 15 mg·L^− 1^ Na_2_SeO_3_ (S5) the MDA content was found to decrease by 21.4% compared with seedlings without PFA (S3). However, with 25 mg·L^− 1^ Na_2_SeO_3_, the MDA content was unchanged after PFA addition (S6) compared with seedlings not treated with PFA (S4) (Fig. 7).

### Effects of PFA on soluble sugar and soluble protein content of peanut seedlings treated with different selenite concentrations

**Fig. 8:**
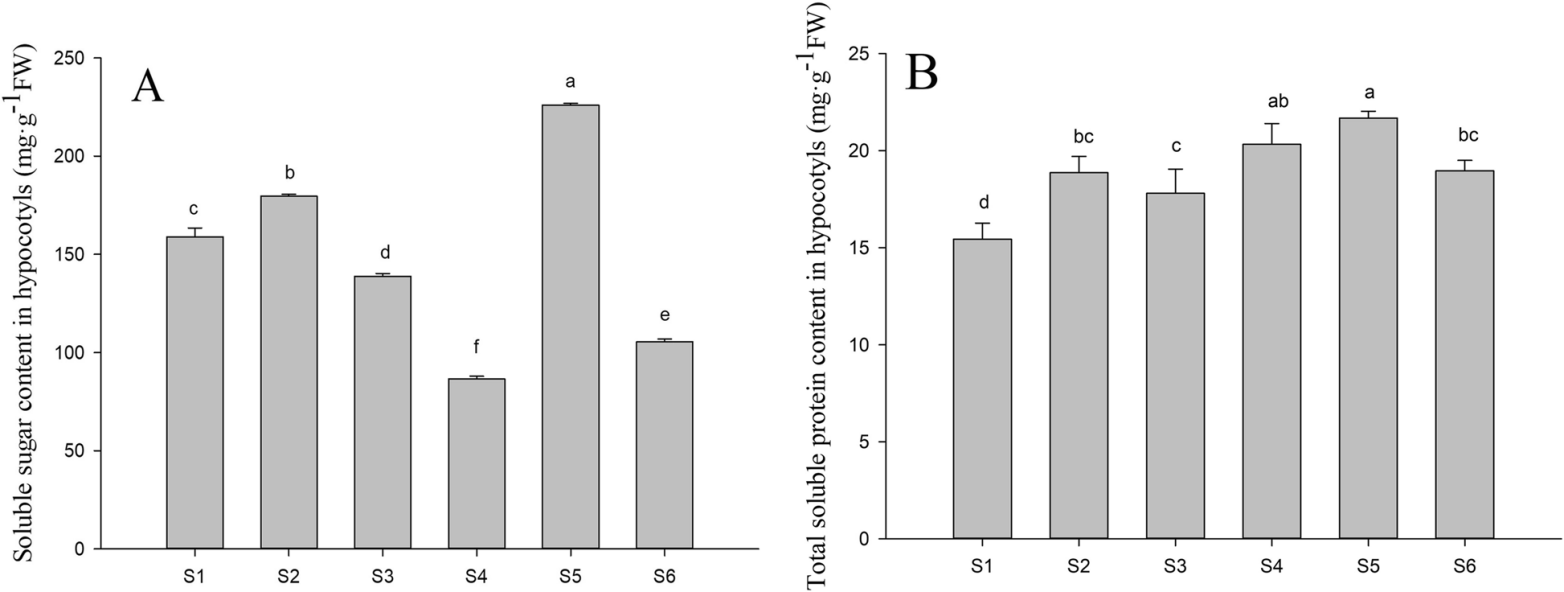
Effects of PFA on soluble sugar and soluble protein contents in hypocotyls of peanut (*Arachis hypogaea* L.) seedlings treated with different selenite concentrations on the 6th day after germination. Letters over the bars indicate significant differences (*P* < 0.05). Vertical bars represent ± SD (*n* = 5). **A** Soluble sugar content. **B** Total soluble protein content in hypocotyls (mg•g-1FW)

As the concentration of Na_2_SeO_3_ rose, the soluble sugar content of the peanut bud hypocotyls increased initially but then decreased (S1-S4). Treatment with 15 mg·L^− 1^ Na_2_SeO_3_ and 25 mg·L^− 1^ Na_2_SeO_3_ increased the soluble sugar content by 62.9 and 21.9%, respectively, after the addition of PFA (S5, S6) compared with before the addition (S3, S4). Surprisingly, the soluble sugar content of the S5-treated seedlings was 42.3% greater than the controls (S1). The soluble protein content in the hypocotyls also increased gradually in response to Na_2_SeO_3_ concentration (S1-S4) with protein concentrations 21.8% greater in the S5 treatment than in the S3 treatment. No significant change in soluble protein content was observed in seedlings treated with 25 mg·L^− 1^ Na_2_SeO_3_ with or without PFA (Fig. 8).

### Effects of PFA on amino acid contents of peanut seedlings treated with different selenite concentrations

**Fig. 9:**
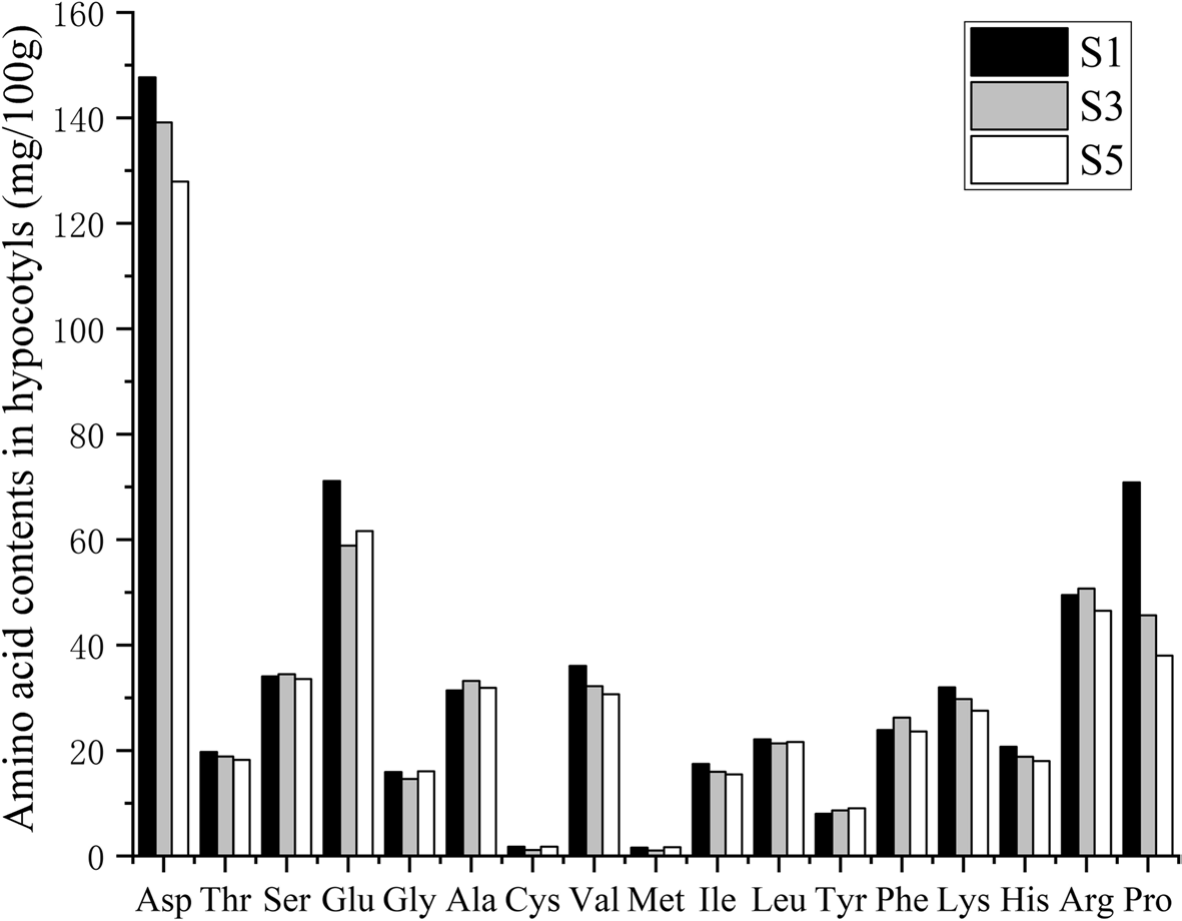
Effects of PFA on amino acid contents in hypocotyls of peanut seedlings treated with different selenite concentrations

The cysteine and methionine contents of peanut bud hypocotyls decreased by 36.0 and 35.0%, respectively, in comparison with S1 when treated with 15 mg·L^− 1^ Na_2_SeO_3_ (S3). However, after the addition of PFA (S5), the cysteine and methionine contents were raised by 57.1 and 65.7%, respectively, compared with S3. The reduction in these amino acids in the hypocotyls of peanut buds can be explained by the mutual competition for sulfur and Se absorption seen in plants [38], leading to reduced sulfur absorption when plants are treated with Se. Se is mostly found in the form of Se-substituted amino acids, usually as a replacement for sulfur. This would lead to reduced concentrations of sulfur in cysteine and methionine. The addition of FA was found to increase both cysteine and methionine contents in the peanut hypocotyls, together with an increase in the overall content of organic Se. In Se-rich peanut seeds, Se is mostly present in the form of protein [30], indicating raised levels of Se-substituted amino acids. This may be because FA treatment increases the contents of cysteine and methionine, to provide sufficient precursors for the synthesis of seleno-amino acids (Fig. 9).

## Conclusions

The processing of black liquor is increasingly important as the demand for paper increases. FA-like compounds extracted from black liquor have can be used for environmentally friendly applications. Here, it was found that, under appropriate Na_2_SeO_3_ concentrations, the addition of PFA significantly increased the total fresh weight of the seedling while decreasing the fresh weight of the root, as well as effectively promoting Se conversion from the inorganic to non-toxic organic forms in the root and hypocotyl, increasing the contents of both soluble sugars and proteins, and thus improving the edible quality and food safety of the Se-enriched peanut buds. This study offers methods for the application of FA-like compounds from black liquor for the production of high-quality Se-enriched peanut sprouts together with the promotion of environmentally friendly uses for paper and pulp by-products.

## Materials and methods

### Isolation of fulvic acid-like compounds from black liquor

Spent liquor was obtained from the Shandong Tranlin Group (Liaocheng, Shandong, China), a wheat-straw processing company. The straw is digested with (NH_4_)_2_SO_3_ and ammonia−water at 240 °C and 0.2 mPa for 400 min. PFAs were isolated as described [39], focusing on PFAs with molecular weights below 10 kDa. PFA concentrations (mg-C/L) were expressed by the organic carbon concentration measured by potassium dichromate oxidation [40].

### Characterization of PFAs

The elemental contents were measured using a Vario EL III (Elementar, Germany) [41]. Fourier-transform infrared spectroscopy (FTIR) was performed on a Nicolet iS10 spectrometer (Thermo Fisher, Waltham, MA, USA) at 400–4000 cm^− 1^ [42].

### Plant materials and treatments

Selected healthy peanut seeds (Yunnan colorful wild ground peanuts) were sterilized in 5% NaClO for 20 min, followed by three to five rinses with sterile water. The seeds were germinated in culture dishes at 20 °C with a sprouting machine (DYJ-S6365, Bear). They were divided into six groups (60 seeds in each group) and sprayed for 1 min once per hour with different concentrations of Na_2_SeO_3_ (0, 5, 15, and 25 mg·L^− 1^ Na_2_SeO_3_; 15 mg·L^− 1^ Na_2_SeO_3_ solution containing 60 mg-C/L PFA; 25 mg·L^− 1^ Na_2_SeO_3_ containing 60 mg-C/L PFA). Following a 6-day growth period, seedlings were sampled to assess relevant changes in physiology or biochemistry.

### Measurement of MDA

After 6 days of seed germination, the MDA content in hypocotyls was determined by the method of Ding et al. (2018) using the formula [43]:

$$\textrm{MDA}\left(\textrm{mmol}\cdot {\textrm{kg}}^{-1}\right)=\frac{\left[6.45\times \left({OD}_{532}-{OD}_{600}\right)-0.56\times {OD}_{450}\right]}{V_s\times m}\times {V}_t\times {V}_r$$

where m is the sample mass, V_r_, V_s_, and V_t_ are the total volume of the reaction solution, the volume of the extract in the solution, and the complete volume of the extract, respectively.

### Enzyme isolation and measurement

One gram of hypocotyls from each treatment condition was homogenized in 3 mL of chilled 50 mM potassium phosphate buffer (pH 7.0) containing 1 mM EDTA, 3 mM β-mercaptoethanol, and 2% (w/v) polyvinylpyrrolidone. After centrifugation (10,000 g, 15 min, 4 °C), the enzyme activities in the supernatants were measured.

Ascorbate peroxidase (APX) was measured as described by Nakano and Asada (1981) using 0.5 mM ascorbate, 0.6 mM H_2_O_2_, and 0.1 mL of enzyme extract in 50 mM potassium phosphate buffer (pH 7.0) in a volume of 3.0 mL. After initiation of the reaction by adding H_2_O_2,_ the reduction in absorbance at 290 nm was determined from 1 to 60 s [44].

Glutathione reductase (GR) activity was determined as described by Knörzer (1996) using a 3 mL reaction solution containing 5 mM MgCl_2_, 0.2 mM NADPH, 1 mM oxidized glutathione (GSSG), and 0.1 mL PFA in 50 mM Tris-HCl, pH 7.5. GSSG was added to initiate the reaction and absorbances at 340 nm were monitored over 60 s [45].

Glutathione S-transferase (GST) activities were determined as described by Habig et al. (1974) [46], Mauch and Dudler (1993) [47]. The 2 mL reaction solution contained 100 μL of glutathione, 800 μL of 1.25 Mm 1-chloro-2,4-dinitrobenzene, and 200 μL PFA in 100 mM PBS, pH 6.5. Increases in absorbance at 340 nm were measured at 30 s intervals and GST activity was calculated as an increase in absorbance of 0.001 per milligram of protein within 1 min.

Peroxidase (POD) activity was assessed as the change in absorbance at 470 nm resulting from the catalysis of H_2_O_2,_ according to Wang (2017) [13]. The reaction solution was prepared by the addition of 112 μL ml guaiacol to 200 ml of PBS (50 mM, pH 6.0), followed by heating and stirring to dissolve it. After cooling to room temperature, 112 μL of 30% H_2_O_2_ was added and mixed well. One hundred microliters of the enzyme extract were added to 2.9 mL of the solution and changes in absorbance at 470 nm were recorded immediately and at 15 s intervals thereafter for 3 min. The enzyme activity was calculated from the absorbance change of 0.01 per milligram of protein within 1 min.

GSH-Px activity was assessed as described by Flohe and Gunzler (1984) [48], Zhang and Wu (2004) [49]. Four hundred microliters of 1.0 mM GSH (containing 2.5 mM NaN_3_) were placed in a 10 mL tube, followed by 400 μL of enzyme solution (blank control was water), and the reaction was initiated by the addition of 200 μL 1.5 mM pre-warmed H_2_O_2_ at 37 °C. After 3 min, 4 mL 1.67% metaphosphoric acid precipitant was added to precipitate the protein and the solution was centrifuged at 3000 rpm for 10 min. Two milliliters of the supernatant were aspirated (blank tube contained 0.4 mL double-distilled water and 1.6 mL 1.67% metaphosphoric acid precipitant) and 2.5 mL of 0.32 M Na_2_HPO_4_ and 0.5 mL DTNB (5,5 ‘- disulfide-p-dinitrobenzoic acid) were added for color development, and the absorbance was measured at 423 nm within 5 min. GSH-Px activity was calculated based on the change of absorbance value of 0.001 per gram of material fresh weight within 1 min (excluding the GSH of non-enzymatic reaction).

### Determination of total soluble sugar content

The total sugar content was determined by the phenol sulfuric acid method described by Dubois (1956) [50]. One gram of the powdered hypocotyls from the different treatments was weighed into a centrifuge tube and 20 mL of double-distilled water was added. The tube was vortexed and placed in a water bath in boiling water for 10 min to fully destroy the cell wall and release the soluble sugars. After centrifugation at 10000 g for 10 min, the supernatant containing the sugar extract was collected, diluted threefold, and 100 μL of the diluent was added to 1 mL of water. Phenol solution (0.5 mL of 6% phenol solution) and concentrated sulfuric acid (2.5 mL) were then added. The solution was mixed well, allowed to stand for 30 min at room temperature, and the absorbance at 490 nm was measured in a spectrophotometer. Standard glucose solutions of different concentrations were prepared in double-distilled water using anhydrous glucose as the reference, and absorbances were read as above. A standard curve was drawn and the total soluble sugar content of the sample was calculated.

### Determination of total soluble protein content

One gram of powdered hypocotyls was added to 3 mL of pre-chilled extraction solution (50 mM potassium phosphate buffer, pH 7.0, containing 1 mm EDTA, 3 mm β- mercaptoethanol, and 2% polyvinylpyrrolidone), vortexed for 1 min, and allowed to stand at 4 °C for 2 h. After centrifugation at 10000 g for 14 min at 4 °C, the total protein content in the supernatant was measured by the Bradford method using bovine serum albumin as the standard [51].

### Determination of organic and inorganic selenium

Six days after the start of selenium enrichment treatment, hypocotyls and roofs were collected from the peanut seedlings, rinsed three to five times with tap water, and blotted to remove the excess water. One gram of sample was used to determine the total and inorganic Se concentrations, as below.

The total Se content was determined according to the national food safety standard of the People’s Republic of China (GB 5009.93–2010, China) for measuring Se in food products. The total Se content was calculated by plotting a standard curve from the data obtained by the Se standard detection.

Measurement of inorganic Se requires sample pretreatment with the addition of 10 mL of 50% hydrochloric acid to the samples in a stoppered test tube. The material was mixed well, ultrasonicated for 30 min, and placed in boiling water for 30 min. After cooling naturally, the material was filtered through absorbent cotton, and the Se-containing filtrate was collected. The Se concentration was measured as described above for the total Se content and was calculated from the standard curve. The organic Se content was calculated as the difference between the total Se content and the inorganic Se content.

### Determination of amino acid content

Two hundred micrograms of hypocotyls were weighed into a hydrolysis tube to which was added 10 mL of 6 M hydrochloric acid. The tube was degassed ultrasonically for 10 min, evaporated under nitrogen, sealed, and placed in an oven at 111 °C for 23 h. The material was then cooled to room temperature, diluted to 100 mL with deionized water, shaken well, and filtered through a 0.45 μm filter. One milliliter was then pipetted into a 10 mL volumetric flask and an aliquot was filtered through a 0.45 μm filter into the injection bottle and analyzed on an L-8900 automatic amino acid analyzer (Hitachi High-Technologies Corp., Tokyo, Japan) [52].

### Statistical analyses

Data were analyzed by SPSS v10.0 using one-way ANOVA with Duncan’s test and significance levels set at *P* < 0.05.

## Data Availability

The datasets used and/or analysed during the current study available from the corresponding author on reasonable request.

## Abbreviations

FA: Fulvic acid
PFA: Fulvic acid-like in paper mill effluents
PBL: Pulp black liquor
MDA: Malondialdehyde
